# Dual core processors: Coupled queues: Transient performance evaluation

**DOI:** 10.1016/j.heliyon.2023.e19059

**Published:** 2023-08-16

**Authors:** Shaik Salma, Garimella Ramamurthy

**Affiliations:** Department of CSE, Mahindra University, Bahadurpalli, 500043, India

**Keywords:** Coupled Queues, Markov Chain, Matrix Exponential, Transient Analysis, Multi-core Processor

## Abstract

High-Performance Computing (HiPC) systems routinely employ multi/many – core processors. Specifically, dual – core processors find many applications in pervasive computing devices. Dual–core processors employ buffers for queueing the incoming jobs. Traditionally, the queues at the processors are assumed to be independent and the queueing system is analyzed in equilibrium for tractability purposes. Queues are modeled using Continuous Time Markov Chains (CTMC's) and the equilibrium performance measures are determined to analyze as well as design the queueing systems. In most interesting cases, the incoming jobs are routed to the queues using the Join the Shortest Queue (JSQ) policy. Thus, with such an adaptive routing algorithm, the two queues are evidently coupled and are not statistically independent. Hence traditional equilibrium performance evaluation doesn't provide realistic performance measures. In this research paper, the two queues associated with buffers in dual-core processors are considered to be coupled. The Coupled Queues are modeled using a Quasi – Birth – and – Death (QBD) process. Using traditional results related to QBD processes, equilibrium performance measures are determined. More interestingly, we demonstrate the tractability of the computation of transient probability distribution of a QBD process. In the research literature, transient analysis of the QBD process was shown to be tractable in the Laplace transform domain. But in this research paper, we prove that the matrix exponential $e^{Qt}$ arising in transient analysis (where *Q* is the generator matrix of the QBD process) can be computed directly in the time domain rendering efficient transient analysis of QBD process. Using the transient probability mass function of queue length, estimation of transient performance measures such as expected queue length, average delay, and tail distribution can be determined. Further, optimal adaptive routing algorithms for coupled queues can be designed.

## 1 Introduction

Computing Systems based on a single processor were utilized in many applications. Multi-Processor (especially multiple cores on a single die) based computing systems provided parallel processing capability [11], thereby enabling high-performance computing. Application-specific processors such as graphics processing units (GPUs) enabled the deployment of versatile computer graphics-based applications. Such hardware accelerators (with hundreds of GPUs) were utilized for general-purpose computing tasks (namely GP GPUs) also.

**Figure 1:**
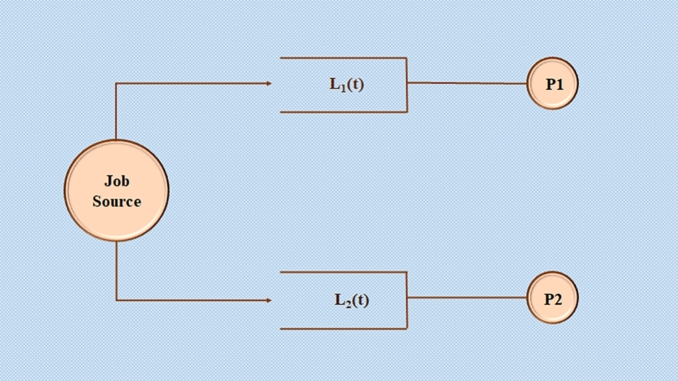
Coupled queues that are statistically dependent.

In most computing platforms (such as multicore processors), buffers are provided to queue the incoming jobs [16]. These buffers could be implemented in software/hardware and the finite buffer size assumption is more reasonable. Specifically, consider a dual-core processor with two buffers and let the incoming computing jobs be routed to the buffers, as shown in Fig. 1.

Most stochastic models of the queueing system assume that the queues are independent. But, most interesting routing algorithms should utilize the queue state information (queue size) for routing the packets to the buffers. Thus, the two queues are not statistically independent and are coupled.

In this research paper, motivated by the application to multicore processors [14], we derive a tractable stochastic model, when there are two queues (The generalization to more than two queues is straightforward) that are coupled.

Strengths:

Traditionally, transient analysis of the QBD process was shown to be tractable [5], [10] in the Laplace transform domain. It requires the additional step of inverse Laplace transform compared to $e^{\mathrm{Qt}}$ computation (8) (where *Q* is the generator matrix).

In this paper, an analytical approach to truncate the matrix power series for $e^{\mathrm{Qt}}$ is provided. This approach works for an arbitrary matrix *Q* (not just a generator matrix). The approach for transient analysis potentially enables real-time computation of transient performance measures of adaptive routing schemes.

## 2 Review of related research literature

In communication networks, to increase throughput and reduce time delay, various adaptive routing, flow control, and congestion control schemes have been proposed [1]. Specifically, in the context of adaptive routing schemes, direct or indirect knowledge of queue lengths is utilized for the flow control of packets. Traditionally Join-the-Shortest Queue is a popular scheme in which packets are routed to the shortest of two queues (picking one of the two alternate paths). Equilibrium performance evaluation of JSQ policy based on stochastic models was proposed in [2], [3], [4]. Optimality of shortest queue routing was established if the length of the queues was continuously monitored.

In optimal, and advanced routing algorithms, the queue length information has to be passed from node to node introducing considerable communication and processing overhead. By transmitting the queue length information infrequently, communication overhead can be reduced. But the information available is out-of-date. Hence, there is a need to make intelligent use of delayed information in adaptive routing algorithms. In order to implement adaptive routing, it is useful to develop a model which enables the prediction of queue length, given an estimate at a prior time. For the purposes of tractability average queue length information conditioned on prior information should be estimated.

In [9], considering the queues to be decoupled and with finite buffer size, an exact closed-form expression for the evolution of queue length distribution is derived.

In [6], we proposed a Quasi-Birth-and-Death (QBD) process model of coupled queues arising in communication networks.

In the following discussion, we provide chronological development of equilibrium and transient analysis of QBD Processes. We explain the motivation for the current approach.

BACKGROUND: Skip-free Markov Chains (e.g., G/M/1 – type Markov Chains, M/G/1 – type Markov Chains) are endowed with geometric recursion for the equilibrium probabilities. i.e.

(1) $$\overset{¯}{\pi }(n+1)=\overset{¯}{\pi }(n)R,$$

where ‘*R*’ (rate matrix) is the matrix solution of the matrix power series equation.

(2) $$\sum_{i=0}^{\infty }R^{i}A_{i}\equiv \overset{¯}{0}$$

(For continuous time skip-free Markov Chains of G/M/1 – type) and in (1), $\overset{¯}{\pi }(n)$ is the equilibrium probability vector of states on level ‘*n*’. In the case of Quasi – Birth and Death (QBD) continuous time Markov Chains, the rate matrix *R* is a matrix solution of a matrix quadratic equation (2), (3).

(3) $$R^{2}A_{2}+RA_{1}+A_{0}\equiv \overset{¯}{0}$$

In the context of level-dependent QBD processes, Latouche et al. showed the existence of matrix geometric recursions of the form.

(4) $$\overset{¯}{\pi }(n+1)=\overset{¯}{\pi }(n)R(n),$$

where rate matrices ($R{\left(n\right)}^{\prime }s$) (4) are solutions of matrix quadratic equations.

Beuermann and Coyle [21] proposed an approach for equilibrium analysis of QBD processes using a state space expansion method (i.e., the number of states at each level are increased) based on the concept of LCI completeness (Level Crossing Information – LCI). In this approach, we have matrix geometric recursion.

(5) $$\overset{¯}{\pi }(n+1)=\overset{¯}{\pi }(n)\overset{¯}{W},$$

where $\overset{¯}{W}$ is the solution of a linear matrix equation and can be computed easily.

Zhang and Coyle [10] were led to the question of whether LCI completeness can be used in the transient analysis of QBD processes. They succeeded in their effort and arrived at a matrix geometric recursion (5) in the Laplace transform (18) domain i.e.

(6) $${\overset{¯}{\pi }}_{\left(n+1\right)}(s)={\overset{¯}{\pi }}_{\left(n\right)}(s)\overset{¯}{W}(s),$$

where $\overset{¯}{W}(s)$ is the solution of the linear matrix equation. (For a level independent QBD Process)

$${\overset{¯}{\pi }}_{\left(n+1\right)}(s)\ldots \text{ Laplace transform of components of vector }{\overset{¯}{\pi }}_{\left(n+1\right)}(t)$$

The transient probability distribution is determined by inverse Laplace transform numerically. Also, with this approach, the number of states at each level can be doubled in the worst case. This approach demonstrated the “feasibility” of transient analysis of QBD processes, arising in applications.

The research reported in this research paper is motivated by the question of whether the transient probability distribution can be “efficiently” computed in the time domain without state space expansion.

Goal: To compute ${\overset{¯}{\pi }}_{n}(t_{0})$ for any specific time value “$t_{0}$” (before the equilibrium is reached) with the time complexity being smaller than by Laplace transform based approach.

## 3 Coupled buffers: Quasi-Birth-and-Death process model

Take a glance at Fig. 1, for an instance of a basic job scheduler [16]. Jobs are routed from the source to the two processors.

### 3.1 Coupled queues: a more precise model

Consider queue length $L_{i}(t)$ (a random variable) available at node *i*. Let the service time be assumed to be exponential with a mean of $1/\mu_{i}$ (for simplicity). At a Poisson rate *λ*, packets arrive at the job source (scheduler) and are routed to either node 1 or node 2.

The source receives the values of $L_{1}(t)$ and $L_{2}(t)$. Based on this information [2], the source directs packets through the intermediary nodes with the shortest queues (it has been demonstrated that if “fresh” information is available, this join-the-shortest-queue approach outperforms the best routing method [13] that ignores queue length information). The paradigm of two independent M/M/1 queues [19] presented in [9], [15], [18] is no longer applicable if the above control is implemented. It is, nonetheless, a useful approximation.

The jobs received at the source are forwarded to the node with the shortest queue length, as previously indicated. If the queue lengths are identical, an independent (statistically) coin toss is done, and the job is routed to either node 1 or node 2. In this scenario, let ‘*p*’ be the probability of routing to queue 1. It is expected that concurrent [20] arrival and departure of jobs [16] from node 1 or node 2 buffers are not permitted (the probability of simultaneous arrival and departure is very small).

Based on the foregoing modeling [12] assumptions, the queues at buffer 1 and buffer 2 are not independent as they have been supposed and utilized in the past.

With the aforementioned modeling assumptions, let us consider $L_{1}(t)$ to be the length of the queue at buffer 1 at time *t* and $L_{2}(t)$ to be the length of the queue at buffer 2 at time *t*. It is easy to see that [$L_{1}(t)$, $L_{2}(t)$] constitutes a finite state space Quasi-Birth-and-Death process. The stochastic model parameters are incorporated in Table 1.

**Table 1:** Parameters in the stochastic model.

S.No	Parameter	Description
1	*μ*	Service rate at both buffers
2	*λ*	Packet arrival rate at source
3	*p*	Probability of selecting queue 1
4	*Q*	Generator Matrix
5	*C*_*N*_	Companion Matrix

### 3.2 Quasi birth and death process generator matrix

Let the size of the buffer at the first node be *N* and the other node also be *N*. As a result, the state space of the level-dependent quasi-birth and death process is defined as follows

E={(i,j):0≤i≤N:0≤j≤N}

The ensuing quasi-birth-death process should be level dependent, based on the modeling assumptions. It will be in the following form.

$$Q=\left(\begin{array}{cccccccc} A_{00} & B_{01} & 0 & 0 & \cdots & \cdots & \cdots & \cdots \\ C_{10} & A_{11} & B_{12} & 0 & \cdots & \cdots & \cdots & \cdots \\ 0 & C_{21} & A_{22} & B_{23} & \cdots & \cdots & \cdots & \cdots \\ \vdots & \vdots & \vdots & \vdots & \cdots & \cdots & C_{NN-1} & A_{NN} \end{array}\right)$$

In the Generator Matrix, each sub–matrix is of the dimension N×N. The sub-matrices in the generator matrix are given in the following description.

$$A_{00}=\left(\begin{array}{cccccccc} -\lambda & (1-p)\lambda & 0 & 0 & 0 & 0 & 0 & 0\\ \mu & -(\mu +\lambda ) & 0 & 0 & 0 & 0 & 0 & 0\\ 0 & \mu & -(\mu +\lambda ) & 0 & 0 & 0 & 0 & 0\\ 0 & 0 & \mu & -(\mu +\lambda ) & 0 & 0 & 0 & 0\\ \cdots & \cdots & \cdots & \cdots & \cdots & \cdots & \cdots & \cdots \\ 0 & 0 & 0 & \cdots & \cdots & \cdots & \mu & -(\mu +\lambda ) \end{array}\right)$$

$$A_{NN}=\left(\begin{array}{cccccccc} -(\mu +\lambda ) & \lambda & 0 & 0 & 0 & 0 & 0 & 0\\ \mu & -2(\mu +\lambda ) & 0 & 0 & 0 & 0 & 0 & 0\\ 0 & \mu & -2(\mu +\lambda ) & \lambda & 0 & 0 & 0 & 0\\ 0 & 0 & \mu & -2(\mu +\lambda ) & \lambda & 0 & 0 & 0\\ \cdots & \cdots & \cdots & \cdots & \cdots & \cdots & \cdots & \cdots \\ 0 & 0 & 0 & \cdots & \cdots & \cdots & \mu & -2\mu \end{array}\right)$$

$$C_{kk-1}=\left(\begin{array}{cccc} \mu & 0 & 0 & \cdots \\ 0 & \mu & 0 & \cdots \\ \cdots & \cdots & \cdots & \cdots \\ \cdots & \cdots & \cdots & \mu \end{array}\right)$$

i.e. a diagonal matrix for k>0. The remaining sub-matrices (11) are as below.

$$B_{kk+1}=\left(\begin{array}{ccccccc} 0 & \cdots & 0 & \cdots & \cdots & \cdots & \cdots \\ \cdots & \cdots & 0 & \cdots & \cdots & \cdots & \cdots \\ 0 & \cdots & 0 & \cdots & \cdots & \cdots & \cdots \\ \cdots & \cdots & \cdots & p\lambda & \cdots & \cdots & \cdots \\ \cdots & \cdots & \cdots & \cdots & \lambda & \cdots & \cdots \\ \cdots & \cdots & \cdots & \cdots & \cdots & \lambda & \cdots \\ \cdots & \cdots & \cdots & \cdots & \cdots & \cdots & \cdots \\ \cdots & \cdots & \cdots & \cdots & \cdots & \cdots & \lambda \end{array}\right)$$

i.e. the $K^{th}$ row and $K^{th}$ column entry is *pλ*. The other higher entries on the diagonal are all $\lambda^{\prime }s$. All the other lower entries in the matrix are zeroes.

$$A_{kk}=\left(\begin{array}{cccccccc} -(\mu +\lambda ) & \lambda & 0 & 0 & \cdots & \cdots & \cdots & 0\\ \mu & -(2\mu +\lambda ) & \lambda & 0 & \cdots & \cdots & \cdots & 0\\ 0 & \mu & -(2\mu +\lambda ) & \lambda & \cdots & \cdots & \cdots & 0\\ 0 & 0 & \mu & -(2\mu +\lambda ) & \lambda & \cdots & \cdots & 0\\ \cdots & \cdots & \cdots & \cdots & \cdots & \cdots & \cdots & \cdots \\ 0 & 0 & \mu & 0 & -(\lambda +2\mu ) & (1-p)\lambda & \cdots & -2\mu \\ \cdots & \cdots & \cdots & \cdots & \cdots & \cdots & \cdots & \cdots \\ \cdots & \cdots & \cdots & \cdots & \cdots & \cdots & \mu & -(\lambda +2\mu ) \end{array}\right)$$

for k≠N,0. Except for the first and the last block matrix on the diagonal i.e. except for $A_{00},A_{NN}$ all the other block diagonal matrices are of the above form i.e. only the ${\left(k+1\right)}^{th}$ row of the block diagonal matrix has the adjacent entries (λ+2μ) and (1−p)λ. The last row has all the entries zero except for the last two entries μ,−(λ+2μ).

### 3.3 Transient performance evaluation

We now discuss the computation of the Transient probability distribution of continuous Time Markov Chains. We first derive the transient probability distribution computation in the Laplace Transform domain.

#### 3.3.1 Algorithm for the computation of transient probability distribution (Laplace transform domain)

It is generally known that the vector-matrix differential equation satisfied by the transient probability distribution of any continuous-time Markov chain [17] (and hence the QBD process), is

(7) $$\frac{d}{dt}\overset{¯}{\pi }(t)=\overset{¯}{\pi }(t)Q,$$

where *Q* is Generator Matrix. The solution to such a differential equation (7) is

(8) $$\overset{¯}{\pi }(t)=\overset{¯}{\pi }(0)e^{Qt}\text{ for }t\geq 0$$

In the case of infinite state space CTMC, $e^{Qt}$ can be computed by the well-known method in control theory. We now provide an approach in the Laplace Transform domain. Applying both sides Laplace transformation (8) [10], we have

(9) $$s\overset{¯}{\pi }(s)-\overset{¯}{\pi }(0)=\overset{¯}{\pi }(s)Q$$

we easily extract the following equation from the above (9) equation.

(10) $$\overset{¯}{\pi }(s)=\overset{¯}{\pi }(0){\left(sI-Q\right)}^{-1}$$

As a result, efficient transient probability distribution computation simply comes down to efficient inversion of the matrix (sI−Q) (10). The Laplace transformation of the transient probability distribution can likewise be gotten recursively (as exhibited in [10]) as shown in the following Lemma.

Notation:

$\overset{¯}{\pi }(t)$: Transient PMF vector at time *t*

$\overset{¯}{\pi }(s)$: Laplace transform vector of $\overset{¯}{\pi }(t)$

Lemma *The Laplace transform of the level dependent quasi birth and death process transient probability vector is obtained recursively using the following recursion.*

(11) $${\overset{¯}{\pi }}_{n}(s)={\overset{¯}{\pi }}_{n+1}(s)W_{n}(s)$$

$W_{n}(s)$ *is computed using the sub-matrices of the generator matrix's.*

Proof Refer [10].

#### 3.3.2 Numerical implementation

Step 1: Using the results in [3], the recursion matrices $W_{n}(s)$ are computed in closed form based on the sub-matrices in the generator matrix.

Step 2: The vectors $\left\{{\overset{¯}{\pi }}_{n}(s\right)$ for n≥0} are determined for several values of ‘*s*’ (say ‘*k*’) (in the region of convergence of the Laplace transform).

Step 3: Using the inverse Laplace transform routine, the vectors $\left\{{\overset{¯}{\pi }}_{n}(t\right)$, n≥0} are determined for several desired values of ‘*t*’.

#### 3.3.3 Transient analysis in the time domain

We now propose a new method for transient probability distribution computation of arbitrary Continuous Time Markov Chains directly in the time domain.

This method is (applies to the Transient analysis of Finite Markov chains (DTMC, CTMC)) based on a novel method of computation of $e^{Qt}$ (7, 8) based on results in [7], [8]. We now briefly summarize the results in [7], [8].

To illustrate the main theorem in [8], we first provide the details in the case of ‘*Q*’ with 2 states. The approach works for an arbitrary 2×2 matrix *X*.

When *X* is a 2×2 matrix, using the Cayley Hamilton theorem, we infer that $X^{j^{\prime }s}$ for j≥2 (with fixed pair of eigenvalues of *X*) can be expressed in terms of matrices *X*, I using scalar coefficients determined by the coefficients of the characteristic polynomial of *X*. For instance, we readily have that

(12) $$X^{3}=(b_{1}^{2}-b_{0})X+(b_{1}b_{0})I$$

Where $Det(\lambda ⁎I-X)=\lambda^{2}+b_{1}\lambda +b_{0}$, with $b_{1}=-Trace(X)$ and $b_{0}=Determinent(X)$ $\left(X^{2}I+b_{1}X+b_{0}I\equiv \overset{¯}{O}\right)$

Now letting,

$$b_{1}^{\left(2\right)}=b_{1},\;b_{0}^{\left(2\right)}=b_{0}\text{ and}\;b_{1}^{\left(3\right)}=(b_{1}^{2}-b_{0}),b_{0}^{3}=b_{1}b_{0}.$$

We have that, the following recursive equation holds:

$$\left(\begin{array}{c} -b_{1}^{\left(3\right)}\\ -b_{0}^{\left(3\right)} \end{array}\right)=\left(\begin{array}{cc} -b_{1}^{\left(2\right)} & 1\\ -b_{0}^{\left(2\right)} & 0 \end{array}\right)⁎\left(\begin{array}{c} -b_{1}^{\left(2\right)}\\ -b_{0}^{\left(2\right)} \end{array}\right)=C_{\left(2\right)}\left(\begin{array}{c} -b_{1}^{\left(2\right)}\\ -b_{0}^{\left(2\right)} \end{array}\right)$$

where $C_{2}$ is a Companion Matrix. This recursion was first observed in [7].

Letting, $X^{4}=-b_{1}^{\left(4\right)}X-b_{0}^{\left(4\right)}I$

It can be readily shown that

$$\left(\begin{array}{c} -b_{1}^{\left(4\right)}\\ -b_{0}^{\left(4\right)} \end{array}\right)={\left(C_{\left(2\right)}\right)}^{2}\left(\begin{array}{c} -b_{1}^{\left(2\right)}\\ -b_{0}^{\left(2\right)} \end{array}\right)$$

In general, $X^{M}$ can be expressed in the terms of (X,I) with two suitable coefficients. The two coefficients are obtained using the following recursion (generalization of the above result):

$$\left(\begin{array}{c} -b_{1}^{\left(M\right)}\\ -b_{0}^{\left(M\right)} \end{array}\right)={\left(C_{\left(2\right)}\right)}^{M-2}\left(\begin{array}{c} -b_{1}^{\left(2\right)}\\ -b_{0}^{\left(2\right)} \end{array}\right)$$

Thus, the coefficients of higher power of *X* can be expressed in terms of a 2×2 companion matrix and the coefficients of characteristics polynomial of *X* (12).

The following theorem is a generalization of the above result and enables efficient computation of $e^{Qt}$ by truncation analytically.

In the following, we consider *Q* instead of *X*. In general, $Q^{m}$, for m≥N can be expressed in terms of $I,Q,Q^{2},Q^{3},...\;Q^{N-1}$ with suitable coefficients. The coefficients can be obtained using the following recursion:

$$\left(\begin{array}{c} -b_{N-1}^{\left(m\right)}\\ -b_{N-2}^{\left(m\right)}\\ -b_{N-3}^{\left(m\right)}.\\ .\\ .\\ -b_{2}^{\left(m\right)}\\ -b_{1}^{\left(m\right)}\\ -b_{0}^{\left(m\right)} \end{array}\right)={\left(C_{N}\right)}^{m-N}\left(\begin{array}{c} -b_{N-1}^{\left(N\right)}\\ -b_{N-2}^{\left(N\right)}\\ -b_{N-3}^{\left(N\right)}\\ .\\ .\\ .\\ -b_{2}^{\left(N\right)}\\ -b_{1}^{\left(N\right)}\\ -b_{0}^{\left(N\right)} \end{array}\right)$$

Where $C_{N}$ is

$$C_{N}=\left(\begin{array}{ccccccccccc} -b_{N-1} & 1 & 0 & 0 & . & . & . & . & 0 & 0\\ -b_{N-2} & 0 & 1 & 0 & . & . & . & . & 0 & 0\\ -b_{N-3} & 0 & 0 & 1 & . & . & . & . & 0 & 0\\ . & . & . & . & . & . & . & . & . & . & \\ . & . & . & . & . & . & . & . & . & . & \\ . & . & . & . & . & . & . & . & . & . & \\ . & . & . & . & . & . & . & . & . & . & \\ -b_{1} & 0 & 0 & 0 & . & . & . & . & 0 & 1\\ -b_{0} & 0 & 0 & 0 & . & . & . & . & 0 & 0 \end{array}\right)$$

i.e. $C_{N}$ is the Companion matrix associated with Characteristic Polynomial of *Q*.

Theorem ${\left(Q\right)}^{m}=-b_{N-1}^{\left(m\right)}⁎Q^{N-1}-b_{N-2}^{\left(m\right)}⁎Q^{N-2}-b_{N-3}^{\left(m\right)}⁎Q^{N-3}....-b_{1}^{\left(m\right)}⁎Q-b_{0}^{\left(m\right)}⁎I$ *. Where Q is* N×N *matrix and* m≥N≥3 *.*

Proof Refer [8].

Now we use the above theorem to compute $e^{Qt}$

(13) $$e^{Qt}=\sum_{j=0}^{\infty }\frac{Q^{j}}{j!}t^{j}$$

By Cayley Hamilton theorem and the above (13) theorem

(14) $$e^{Qt}=\;\sum_{j=0}^{N-1}\frac{Q^{j}t^{j}}{j!}+\sum_{j=N}^{\infty }\left(-\sum_{i=0}^{N-1}b_{i}^{\left(j\right)}Q^{i}\right)\frac{t^{j}}{j!}$$

Changing indices in the second summation (14)

(15) $$e^{Qt}=\;\sum_{j=0}^{N-1}\left[\frac{Q^{j}t^{j}}{j!}\right]-\sum_{j=0}^{N-1}\left[\sum_{i=N}^{\infty }b_{j}^{\left(i\right)}Q^{j}\frac{t^{i}}{i!}\right]$$

$$e^{Qt}=\sum_{j=0}^{N-1}\left[\frac{Q^{j}t^{j}}{j!}\right]-\sum_{j=0}^{N-1}\left[\sum_{i=N}^{\infty }\frac{b_{j}^{\left(i\right)}t^{i}}{i!}\right]Q^{j}$$

We readily have that $\overset{¯}{\pi }(t)=\overset{¯}{\pi }(0)e^{Qt}$, where $\overset{¯}{\pi }(t)$ is the vector of transient probabilities of a CTMC and $\overset{¯}{\pi }(0)$ is the initial probability vector.

It should be noted that by theorem, the coefficients $b_{j}^{{\left(i\right)}^{\prime }}s$ can be (15) computed recursively.

Using the transient probability mass function, various time-varying performance measures can be readily computed. Some of them are directly related to the moments of the transient PMF.

#### 3.3.4 Efficient computation of transient probability distribution of CTMC

(16) $$e^{Qt}=\;\sum_{j=0}^{N-1}\left[\frac{Q^{j}t^{j}}{j!}\right]-\sum_{j=0}^{N-1}\left[\sum_{i=N}^{\infty }\frac{b_{j}^{\left(i\right)}t^{i}}{i!}\right]Q^{j}$$

Using the recursion for the coefficients $b_{j}^{{\left(i\right)}^{\prime }}s$.

I.e. ${\overset{¯}{b}}^{\left(i\right)}=\overset{¯}{C}{\overset{¯}{b}}^{\left(i-1\right)}$ (for i≥N), the inner summation in the above (16) expression is truncated (e.g. when $L^{2}-norm\;({\overset{¯}{b}}^{\left(i\right)})$ is sufficiently small). Thus, we have

(17) $$e^{Qt}=\;\sum_{j=0}^{N-1}Q^{j}\left(\frac{t^{j}}{j!}-\sum_{i=N}^{L}b_{j}^{\left(i\right)}\frac{t^{i}}{i!}\right)$$

In the above expression (leading to transient PMF), {$Q^{j}$ for j≥2} can be recursively computed. We may be interested in computing $e^{Qt}$ at integer multiples of say “$t_{0}$”.

Thus $\left\{e^{Qt}:t\in \{t_{0},2t_{0},\ldots \}\right\}$ is precomputed using the above (17) approximation. Using such approximation transient PMF and hence the transient performance measures are determined at integer multiples of times “$t_{0}$”.

## 4 Numerical results

A specific example is used in the following discussion to demonstrate the efficient computation of equilibrium distribution.

At Node 1 and Node 2, the Max buffer length is 2

Service rate at both nodes is μ=0.5

Packet arrival rate at source is λ=0.3

Probability of selecting queue 1 when both have the same size is p=0.6

The following description of the generator's sub-matrices is given with these parameters. They are inferred from the sub matrix general expressions in Section 3. However certain sub-matrices are included here for clarification.

$$Q=\left(\begin{array}{ccc} A_{00} & B_{01} & 0\\ C_{10} & A_{11} & B_{12}\\ 0 & C_{21} & A_{22} \end{array}\right)$$

For the above values of *λ* and *μ*, the sub-matrices are given by

$$A_{00}=\left(\begin{array}{ccc} -0.3 & 0.12 & 0\\ 0.5 & -0.8 & 0\\ 0 & 0.5 & -0.8 \end{array}\right)$$

$$A_{11}=\left(\begin{array}{ccc} -0.8 & 0.3 & 0\\ 0.5 & -1.3 & 0.12\\ 0 & 0.5 & -1.3 \end{array}\right)$$

$$A_{22}=\left(\begin{array}{ccc} -0.8 & 0.3 & 0\\ 0.5 & -1.3 & 0.3\\ 0 & 0.5 & -1 \end{array}\right)$$

$$B_{01}=\left(\begin{array}{ccc} 0.18 & 0 & 0\\ 0 & 0.3 & 0\\ 0 & 0 & 0.3 \end{array}\right)$$

$$B_{12}=\left(\begin{array}{ccc} 0 & 0 & 0\\ 0 & 0.18 & 0\\ 0 & 0 & 0.3 \end{array}\right)$$

$$C_{10}=\left(\begin{array}{ccc} 0.5 & 0 & 0\\ 0 & 0.5 & 0\\ 0 & 0 & 0.5 \end{array}\right)$$

$$C_{21}=\left(\begin{array}{ccc} 0.5 & 0 & 0\\ 0 & 0.5 & 0\\ 0 & 0 & 0.5 \end{array}\right)$$

Now we will compute the values of $I_{0},I_{1}$ and $I_{2}$ recursively.

$$I_{0}=A_{00}=\left(\begin{array}{ccc} -0.3 & 0.12 & 0\\ 0.5 & -0.8 & 0\\ 0 & 0.5 & -0.8 \end{array}\right)$$

$$I_{1}=A_{11}-C_{10}I_{0}^{-1}B_{01}=\left(\begin{array}{ccc} -0.4 & 0.4 & 0\\ 0.75 & -1.05 & 0.12\\ 0.1563 & 0.6563 & -1.1125 \end{array}\right)$$

$$I_{2}=A_{22}-C_{21}I_{1}^{-1}B_{12}=\left(\begin{array}{ccc} -0.8 & 0.7238 & 0.0762\\ 0.5 & -0.8762 & 0.3762\\ 0 & 0.8095 & -0.8095 \end{array}\right)$$

$\pi_{2}$, denotes the equilibrium probability of states on level ‘*i*’.

Starting with ${\overset{¯}{\pi }}_{2}$, the equilibrium probabilities are recursively computed and normalized.

$$\pi_{0}=-\pi_{1}C_{10}I_{0}^{-1}=\left(\begin{array}{ccc} 0.5330 & 0.1390 & 0.0085 \end{array}\right)$$

$$\pi_{1}=-\pi_{2}C_{21}I_{1}^{-1}=\left(\begin{array}{ccc} 0.1808 & 0.0860 & 0.0136 \end{array}\right)$$

$$\pi_{2}=null(I_{2})=\left(\begin{array}{ccc} 0.0114 & 0.0182 & 0.0095 \end{array}\right)$$

null$\left(I_{2}\right)$ is the left null space of matrix $I_{2}$.

Using the transient analysis methods, the transient PMF and transient performance measures are computed.

Zhang and Coyle approach requires state space expansion which requires doubling the number of states at each level (in the worst case). For 100 values of ‘*s*’, the accuracy of the Laplace transform approach and our approach is the same. As ‘*s*’ increases, the accuracy has increased for any value of ‘*t*’ under transient conditions.

For each value of ‘*s*’, a matrix inversion is needed whose time complexity is $O(M^{3})$ Where ‘*M*’ is the number of states at each level after state space expansion (to ensure LCI completeness). Also, one matrix multiplication is required to compute W(S).

To be able to determine the transient probability mass function over a finite time horizon (till equilibrium is attained), ${\overset{¯}{W}}_{n}(s)$ needs to be computed for a large, finite number of values of ‘*s*’. Also, the inverse Laplace transform needs to be computed to determine.

$$\overset{¯}{\pi }(t)\text{ for }t\geq 0\text{ (till the equilibrium reached)}$$

Furthermore, since the QBD model (of coupled queues) is level dependent, a recursion of the form.

(18) $${\overset{¯}{\pi }}_{\left(n+1\right)}(s)={\overset{¯}{\pi }}_{\left(n\right)}(s){\overset{¯}{W}}_{n}(s)$$

Thus, the recursion matrix, ${\overset{¯}{W}}_{n}(s)$ is also level dependent, and it needs to be computed for each level ‘*n*’. This increases the time complexity of the algorithm (for transient analysis based on the Laplace transform-based approach (6) of Zhang and Coyle).

Thus, the time complexity of such an approach depends on

Number values of ‘*s*’ for which ${\overset{¯}{W}}_{n}(s)$ is evaluated.

Also, computations involved in determining the inverse Laplace transform will further increase the time complexity. The exact determination of time complexity is avoided for the sake of brevity.

Summary: The time Complexity of the Laplace transform approach is much larger than that of our approach.

In the adaptive routing, the parameters *λ*, *μ* potentially change. In our approach, we only compute transient PMF for a single time instant, say ‘$t_{0}$’.

The following plots illustrate the transient analysis of coupled queues arising in dual-core processor performance evaluation. Table 2 explicitly describes the transient evolution of $\pi_{\left(0,1\right)}(t)$, $\pi_{\left(1,1\right)}(t)$, E[X(t).

**Table 2:** Transient evolution of probabilities and expected value.

S.No	Time (Sec)	*π*_(0,1)_(*t*)	*π*_(1,1)_(*t*)	*E*[*X*(*t*)]
1	0	0.31	0	0
2	0.17	0.26	0.075	0.9
3	0.27	0.21	0.101	0.11
4	0.37	0.18	0.103	0.115
5	0.47	0.16	0.1	0.117
6	0.74	0.15	0.09	0.15

We now briefly summarize the information in the following graphs related to transient performance evaluation.

**Figure 2:**
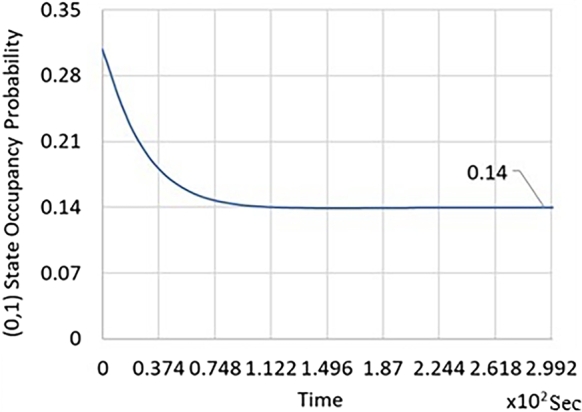
Time dependent probability evolution for QBD that starts in (0,1) state with probability 0.31 (i.e. Queue 2 has one job in the queue at time 0 and no jobs in queue 1).
• In Fig. 3, with zero probability that queues 1 and 2 have even one job; the transient evolution of $\pi_{\left(1,1\right)}(t)$ is depicted.

• In Fig. 2, with one job in Queue 2 and no jobs in Queue 1 initially, it is shown how the transient probability reaches the equilibrium probability.

**Figure 3:**
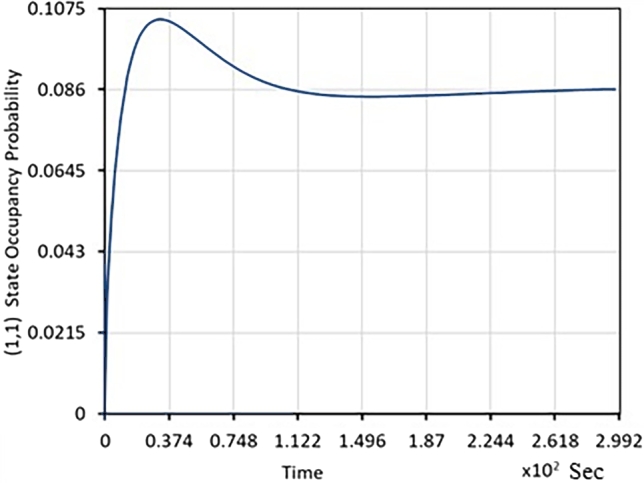
Probability as a function of time that the process is in the state (1,1). (At time 0, the probability that queues 1 and 2 have one job is zero.).
• In Fig. 4, with zero average queue length at Queue 1, the time evolution of mean queue length is shown.

**Figure 4:**
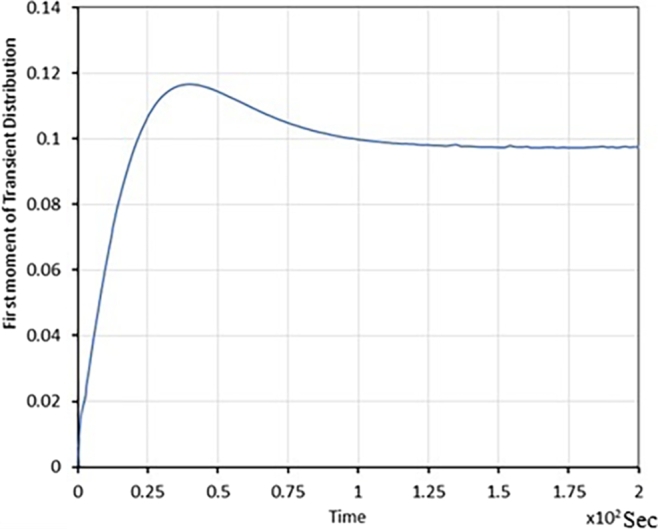
Time varying Average Queue length of Queue, 1 (for different initial conditions).
• In Fig. 5, the time-dependent probability that queue length in Queue 1 is more than 1 is illustrated.

**Figure 5:**
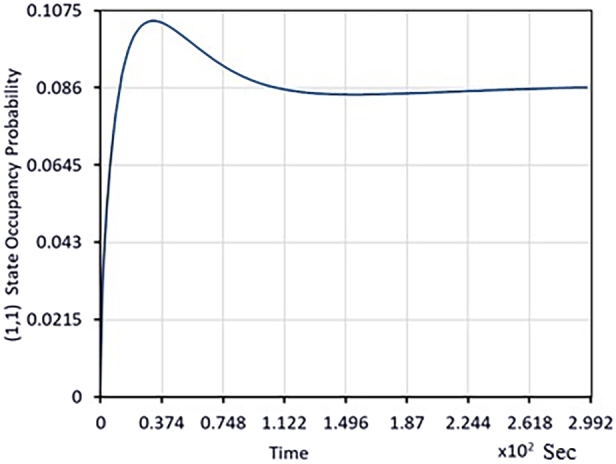
The tail distribution of QBD Modeling coupled queues (probability that queue length in queue 1 is more than 1).

From Figure 2, Figure 3, Figure 4; the relevant important information is summarized in Table 2.

### 4.1 Utility of methods for determining the transient probability distribution

The Laplace transformation-based approach is utilized when the transient probability distribution is determined over a wide time horizon (including the convergence to the equilibrium probability distribution).

The time domain approach is preferred when transient probability distribution needs to be determined probability for few (mostly one) time instances.

## 5 Conclusion

In this research paper, a time domain approach to the transient analysis of arbitrary, finite state space CTMC is proposed. It is computationally more efficient than Laplace transform (S - domain) domain approach. Also, by modeling queues arising in dual-core processors as being coupled, efficient transient performance evaluation is demonstrated.

The proposed approach doesn't directly lead to real-time computation of transient performance measures. We hope to investigate real-time transient performance evaluation in future work. Generalizations of the stochastic model for applications in high-performance computing systems and communication networks are proposed for future work.

## CRediT authorship contribution statement

Garimella Ramamurthy: Conceived and designed the experiments; Analyzed and interpreted the data.

Shaik Salma: Performed the experiments; Contributed reagents, materials, analysis tools, or data; Wrote the paper.

## Declaration of Competing Interest

The authors declare that they have no known competing financial interests or personal relationships that could have appeared to influence the work reported in this paper.

## Data Availability

Data will be made available on request.
